# RNA-triggered innate immunity: friend and foe

**DOI:** 10.3389/fgene.2026.1775625

**Published:** 2026-02-23

**Authors:** Maike Henschel, Maria R. Conte, Rocio T. Martinez-Nunez

**Affiliations:** 1 Randall Centre for Cell and Molecular Biophysics, School of Basic and Medical Biosciences, King’s College London, London, United Kingdom; 2 Department of Infectious Diseases, School of Immunology and Microbial Sciences, King’s College London, London, United Kingdom

**Keywords:** host vs pathogen, immune pathology, innate immunity, mRNA therapeutics, RNA modifications, RNA sensing

## Abstract

Endogenous, or *‘self’*, vs. microbial, or *‘non-self’*, RNA sensing can tip the scales between immune pathology and effective immunity. Cells are equipped to sense RNA, fundamental to trigger an innate immune response to clear viral infection that should not generate a harmful immune response against endogenous RNA. Multiple chemical modifications in RNA fine-tune its cellular sensing and are exploited by pathogens to evade immunity. Likewise, perturbations triggering self RNA sensing cause immune pathologies. This underscores the clinical need for a better understanding of self RNA recognition. Here, we address nucleic acid sensing in the innate immune response from an RNA-centric view. We discuss how self RNA is shielded from sensing by chemical modifications and subcellular compartmentalization, possible mechanisms and consequences of self-RNA sensing, and how this knowledge has been harnessed to revolutionize vaccine development.

## 1 Introduction

RNA is one of the most versatile cellular macromolecules: besides its best-known function as a transcript of DNA and template for protein translation (Brenner et al., 1961), RNA is part of cellular structures (Palade, 1955), possesses enzymatic capabilities in ribozymes (Noller et al., 1992), and can fine-tune gene expression (Brown et al., 1991). RNA is a major immunostimulatory molecule, as viral RNA genomes and replication intermediates can be recognized by and activate the innate immune system (Luan et al., 2024). The ability to sense exogenous nucleic acids is conserved from bacteria to humans (Linn and Arber, 1968; Yoneyama et al., 2004; Barrangou et al., 2007; Ledvina and Whiteley, 2024), making it the most ancient mechanism of immunity against pathogens. Yet this fundamental defense mechanism comes with a major challenge: with 10^7^ endogenous RNA molecules in any mammalian cell (Palazzo and Lee, 2015), cells must constantly distinguish their own RNA from exogenous RNA. RNA sensing thus strikes a fine balance, preventing a harmful inflammatory response to endogenous, or *‘self’*, RNA while allowing recognition of, and effective defense against, exogenous, or *‘non-self’*, RNA.

Incorrect self RNA sensing has pathological consequences, including chronic inflammation, type I interferonopathies, and autoimmunity (Crow and Stetson, 2022; Li et al., 2024). These immune disorders have profound systemic effects and are in many cases lethal. For example, gene mutations inducing endogenous RNA sensing can cause Aicardi-Goutières syndrome (AGS) (Rice et al., 2012; Rice et al., 2014), an inflammatory condition with high childhood mortality rates, where interferon (IFN) overproduction leads to brain injury (Liu and Ying, 2023). Conversely, agonists activating cellular RNA sensors are used to enhance immunotherapies (Frega et al., 2020). This underscores the clinical need for a better understanding of self RNA recognition to minimize its sensing, avoid detrimental outcomes, and explore its potential as an adjuvant.

While the contributions of nucleic acid sensors in preventing endogenous RNA sensing have been reviewed elsewhere (Rehwinkel and Gack, 2020; Shimizu, 2024), the significance of contributions from the RNA perspective is underappreciated. In this mini-review, we explore nucleic acid sensing from an RNA-centric view. Specifically, we discuss compartmentalization and RNA modifications as central mechanisms preventing self RNA sensing, highlight how failure of safeguarding mechanisms contributes to immune pathology, and how a deeper understanding of self vs. non-self RNA sensing can contribute to the development of mRNA-based therapeutics.

## 2 Cellular RNA sensors: antiviral sentinels

**FIGURE 1:**
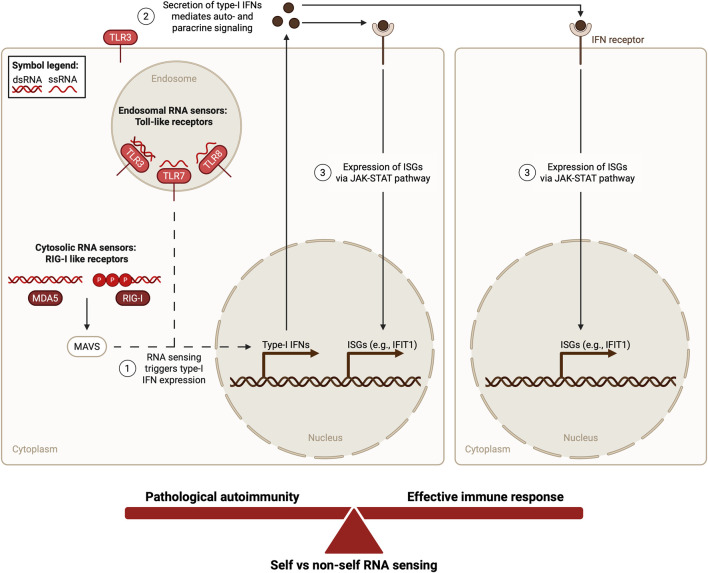
Overview of cellular RNA-sensing pathways. Cells are equipped with endosomal RNA sensors, Toll-like receptors (TLRs), and Cytosolic RNA sensors, RIG-I-like receptors (RLRs). TLR3 senses double-stranded RNA (dsRNA), while TLR7 and TLR8 sense single-stranded RNA (ssRNA). The RLRs RIG-I and MDA5 have a stronger affinity for dsRNA than ssRNA. MDA5 detects longer dsRNAs, while RIG-I is activated by shorter dsRNA structures and uncapped 5′ triphosphate ends. RNA sensing via TLRs and RLRs triggers a signaling cascade leading to the transcription of type-I interferons (IFNs). Secretion of type-I IFNs leads to autocrine and paracrine signaling, inducing interferon-stimulated gene (ISG) expression via the JAK-STAT pathway. This mechanism needs to be highly regulated to prevent a harmful inflammatory response against endogenous RNA while allowing recognition of, and effective defense against, exogenous RNA, thereby striking a balance between effective immune defense and pathological autoimmunity. Created in BioRender (https://BioRender.com/5wibkrr).

Cells are equipped with pattern recognition receptors (PRRs), which recognize viral RNAs as pathogen-associated molecular patterns (PAMPs), triggering an innate immune response crucial for viral clearance (Medzhitov and Janeway, 2000). Similarly, PRRs are capable of sensing endogenous RNAs released from damaged or dying cells as damage-associated molecular patterns (DAMPs) (Man and Kanneganti, 2024). Analysis of the expression of endosomal Toll-like receptors (TLRs) that detect RNA shows that they are expressed in multiple cell types (Lonsdale et al., 2013) beyond their enrichment in immune cells (Takaoka and Yamada, 2019). Double-stranded RNA (dsRNA), such as viral replication intermediates, is detected by TLR3 and single-stranded RNA (ssRNA) via TLR7 and TLR8 (Alexopoulou et al., 2001; Diebold et al., 2004; Heil et al., 2004; Greulich et al., 2019; Asami and Shimizu, 2021). The ubiquitously expressed RIG-I-like receptors (RLRs), retinoic-acid inducible gene-I (RIG-I) and melanoma differentiation-associated protein 5 (MDA5), sense dsRNA, and with less affinity ssRNA, in the cytosol (Yoneyama et al., 2004; Kato et al., 2008; Takahasi et al., 2008; Takaoka and Yamada, 2019). RIG-I and MDA5 function is enhanced by laboratory of genetics and physiology 2 (LGP2), another RLR (Satoh et al., 2010). RNA sensing by either TLRs or RLRs triggers a downstream antiviral response, which includes the expression of type I IFNs and pro-inflammatory cytokines (Takeuchi and Akira, 2007). Type I IFN secretion triggers expression of IFN-stimulated genes (ISGs) through autocrine and paracrine signaling (Figure 1). Many ISGs act as viral replication restriction factors and also possess nucleic acid-sensing capabilities, such as the IFN-induced proteins with tetratricopeptide repeats (IFITs), which detect ssRNAs and inhibit their translation (Pichlmair et al., 2011). Similarly, the ISGs Z-DNA binding protein 1 (ZBP1) and protein kinase R (PKR) sense nucleic acids, triggering programmed cell death and global translation inhibition, respectively (Zhang et al., 2001; Thapa et al., 2016). While RNA sensing is therefore crucial for an adequate immune response against pathogens, a fine balance is required to avoid immunity triggered via endogenous RNA, putting self vs. non-self RNA recognition between effective immune defense and pathological autoimmunity (Figure 1). With so many opportunities for self RNA sensing, how do cells maintain homeostasis? We present two main mechanisms: subcellular compartmentalization and disguising with chemical modifications.

## 3 Compartmentalization of RNA sensors and immunostimulatory RNA: preventing pathological innate immunity

RNA and RNA-sensors separate into different subcellular compartments to avoid self-triggered inflammation. TLRs localize in endosomes (Lande et al., 2007; Lind et al., 2022), where they encounter RNAs of viruses entering through the endocytic pathway (Jensen and Thomsen, 2012) and where endogenous RNA is absent.

**FIGURE 2:**
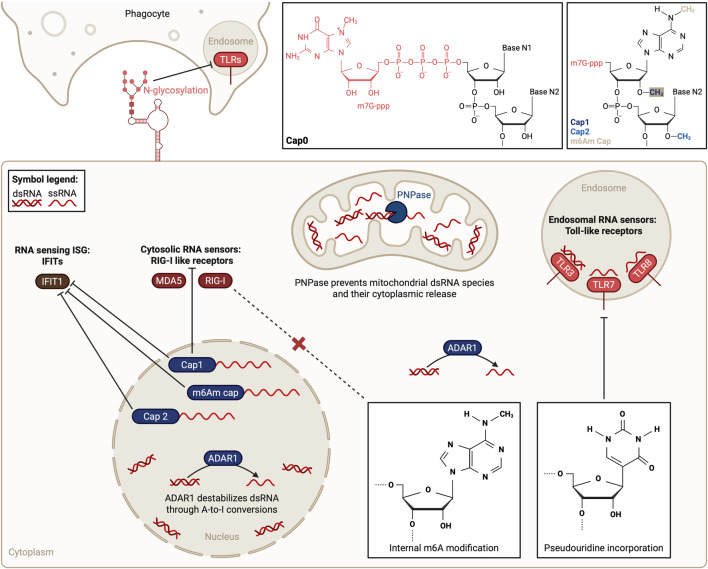
Overview of protection mechanisms preventing self RNA sensing. Endosomal Toll-like receptor (TLR) localization prevents sensing of cellular RNAs not found in endosomes. Sequestering immunostimulatory double-stranded RNA (dsRNA) species to the mitochondria through PNPase activity and the nucleus limits cytosolic dsRNA levels, preventing their sensing. Moreover, adenosine (A) to inosine (I) conversions catalyzed by ADAR1 destabilize dsRNA. Nuclear 2′-O-ribose methylation, forming Cap1 and Cap2 structures, impedes RLR and IFIT1 stimulation. Additional N6-methyladenosine (m6Am) modification of Cap1 further decreases RNA recognition by IFIT1. Internal m6A modifications prevent RNA sensing by RIG-I. Pseudouridine incorporation into RNAs and N-glycosylation of small cell surface RNAs prevent TLR stimulation in monocyte-derived dendritic cell experiments and during efferocytosis, respectively. Blunt-ended arrow indicates direct prevention of PRR activation. Dotted arrow indicates indirect effect. Created in BioRender (https://BioRender.com/z5ktf44).

Potentially immunostimulatory endogenous RNAs are also compartmentalized to prevent cytosolic RLR binding. dsRNA is the main target of RLRs, making it more immunostimulatory than ssRNA (Rehwinkel and Gack, 2020). While RNA is generally thought of as a single-stranded molecule, dsRNA species can arise due to bidirectional (forward and reverse) transcription of the nuclear and mitochondrial genomes (Kapranov et al., 2007). Nuclear retention of dsRNA species thus minimizes sensing by cytosolic PRRs (Sadeq et al., 2021). However, most detectable cellular dsRNA is of mitochondrial origin (Dhir et al., 2018). Mitochondria possess a circular genome formed of a heavy and a light strand (Chinnery and Hudson, 2013). Bidirectional transcription of mitochondrial DNA generates overlapping transcripts that can form immunostimulatory dsRNA structures (Gustafsson et al., 2016), which are suppressed through degradation of light strand transcripts by enzymes such as polynucleotide phosphorylase (PNPase) (Borowski et al., 2013), preventing sensing by cytoplasmic PRRs (Figure 2). Accordingly, PNPase depletion leads to increased levels and cytoplasmic localization of mitochondrial dsRNA, triggering an MDA5-driven IFN response (Dhir et al., 2018). Such sensing can lead to pathology, as individuals with decreased PNPase levels show higher cytoplasmic accumulation of dsRNA, making them susceptible to aberrant immune activation (Dhir et al., 2018).

## 4 RNA modifications are molecular safeguards against self RNA sensing

In addition to mitochondrial RNA, many other subtypes of RNAs such as ribosomal RNA (rRNA) and transfer RNA (tRNAs) are found in the cytoplasm and subject to RLR detection. Hence, subcellular separation of RNA and PRRs insufficiently explains prevention of self RNA sensing. Alternative concepts were explored by the two Nobel laureates Katalin Karikó and Drew Weissman. Methylation of CpG motifs, a major epigenetic mark, was at the time shown to prevent DNA sensing (Hemmi et al., 2000). Karikó and Weissman investigated whether RNA modifications could similarly dampen self-RNA sensing. They demonstrated that the quantity of RNA modifications depended on RNA subtype and correlated directly with the evolutionary level of the organism (Karikó et al., 2005). Mammalian RNAs bore more modifications than bacterial RNAs, minimizing mammalian RNA immunogenicity (Karikó et al., 2005). We now estimate that RNA undergoes over 170 different nucleoside modifications (Sordyl et al., 2026).

In hallmark experiments, Karikó et al. compared the immunostimulatory potential of bacterial RNA, *in vitro* transcribed RNA, and different endogenous RNA species by transfecting them into monocyte-derived dendritic cells (MDDCs). The degree of RNA modifications negatively correlated with their immunogenic potential. For example, mammalian tRNAs, the most heavily modified subgroup of endogenous RNAs, were non-immunostimulatory. In contrast, mitochondrial RNA, like bacterial RNA, had fewer RNA modifications and was more immunogenic than other cellular RNAs. In other words, the authors concluded that an increasing amount of nucleoside modifications prevented MDDCs stimulation by RNA (Karikó et al., 2005). Here, we will review the most studied endogenous chemical RNA modifications and base changes preventing self RNA sensing-induced innate immunity. As a given RNA transcript contains diverse RNA modifications, it remains a challenge and open question to distinguish their respective contribution to immune tolerance.

### 4.1 The beginning: pseudouridine incorporation

Karikó and Weissman explored the potential of RNA modifications to dampen immunogenicity by incorporating different modified ribonucleotides into *in vitro* transcribed RNAs (Karikó et al., 2005). Pseudouridine incorporation repressed the ability of RNA to stimulate TLR7 and TLR8, abolishing RNA-mediated activation of MDDCs (Karikó et al., 2005) (Figure 2). Specifically, pseudouridine-incorporation impairs RNA processing by endolysosomal nucleases and RNAs can no longer act as TLR agonists. In addition, pseudouridine also directly prevents RNA binding to TLR7 and TLR8 (Bérouti et al., 2025). Pseudouridine incorporation allowed the generation of nonimmunogenic *in vitro* transcribed messenger RNAs (mRNAs) (Karikó et al., 2008). This fundamental finding served as a basis to create mRNA vaccines that do not trigger a harmful IFN response upon cellular uptake, such as the SARS-CoV-2 mRNA vaccines (Verbeke et al., 2021). Karikó’s and Weissman’s research not only laid the groundwork for the manufacturing of mRNA-based therapeutics but also revealed a novel self RNA recognition protection mechanism: RNA modifications.

### 4.2 Choosing the right cap: 2′-O-ribose methylation

Cellular mRNAs present a 5′ cap, enhancing their stability and translation and shielding them from PRR recognition. The 5′ cap protects from RIG-I binding, which is activated by uncapped 5′ triphosphate RNA ends (Hornung et al., 2006), present in viral RNAs. Cellular pre-mRNA transcribed in the nucleus is processed by removal of the free 5′ triphosphate ends and addition of a 5′ N7-methylguanosine (m7G) cap, forming the Cap0 structure (Shatkin, 1976). RIG-I can also bind dsRNA equipped with a Cap0 structure, leading to a downstream IFN response (Devarkar et al., 2016). 2′-O-ribose methylation, i.e., addition of a methyl group to the 2′-hydroxyl of the ribose sugar of the first and second nucleotides of the m7G cap, generates the Cap1 and Cap2 structures, respectively. Cap1 can abolish binding to RIG-I (Schuberth-Wagner et al., 2015; Devarkar et al., 2016) and MDA5 (Züst et al., 2011) (Figure 2).

Additionally, Cap1 prevents RNA recognition by the ISG IFIT1 (Figure 2), which binds mRNAs with Cap0 structures, inhibiting their translation (Habjan et al., 2013; Abbas et al., 2017). During infection, the mRNA cap methyltransferase 1 (CMTR1) regulates expression of ISG mRNAs by promoting Cap1 formation, shielding them from IFIT1 recognition and subsequent translation inhibition (Williams et al., 2020). Thus, 2′-O-ribose methylation contributes to an effective innate immune response by preventing Cytosolic self RNA sensing, selectively protecting cellular antiviral RNAs from degradation or translation inhibition during a viral infection. As viral infections can trigger widespread RNA degradation, this limits the opportunity for self-sensing (Glaunsinger et al., 2005; Burke et al., 2021a; Burke et al., 2021b).

### 4.3 The allrounder: N6-methyladenosine

N6-methyladenosine (m6A) modification can also shield RNAs from sensing and can be classified into cap-adjacent and RNA internal m6A modifications. Cap1-adjacent m6A (m6Am) modification is important for preventing IFIT1 RNA binding (Figure 2), protecting endogenous mRNAs from translation inhibition (Habjan et al., 2013). Specifically, m6Am modification but not internal m6A modification of the 5′ untranslated region blocked IFIT1 binding more strongly than Cap1 alone (Geng et al., 2024). Internal m6A modification of *in vitro* transcribed mRNAs reduced their immunostimulatory potential (Karikó et al., 2005) and is utilized by cells and viruses. For example, m6A incorporation by METTL3 reduces the formation of immunostimulatory endogenous dsRNA species during hematopoietic development (Gao et al., 2020) but viruses exploit it to avoid immune detection (Lichinchi et al., 2016; Kennedy et al., 2017; An et al., 2020; Li et al., 2021; Qiu et al., 2021). For example, METTL3 decreases viral dsRNA formation by m6A modification of vesicular stomatitis virus RNA (Qiu et al., 2021), preventing RLR activation. Additionally, m6A-modified nucleotides on viral RNAs are bound by m6A readers, blocking RIG-I binding (Kim et al., 2020; Lu et al., 2020) (Figure 2). Thus, a better understanding of self RNA recognition can deliver fundamental information on viral immune escape pathways, enabling drug development capable of treating viral infections.

### 4.4 Sweet RNA: N-glycosylation

Recently, it has been identified that in addition to proteins and lipids, RNAs can also undergo glycosylation (Flynn et al., 2021), revealing a novel class of RNA modifications previously restricted to monosaccharide-based tRNA modifications (Kasai et al., 1976; Okada et al., 1977). Specifically, small noncoding RNAs can be modified with sialic acid-containing N-linked glycans (glycoRNAs), the majority of which localize to the cell surface (Flynn et al., 2021). The N-glycosylation is attached at the modified RNA base 3-(3-amino-3-carboxypropyl) uridine (acp^3^U) site (Xie et al., 2024).

The presence of cell surface glycoRNAs poses a challenge for the non-inflammatory (i.e., immunologically silent) clearance of apoptotic cells. Apoptotic cells are removed by phagocytic cells in a non-inflammatory process called efferocytosis (Arandjelovic and Ravichandran, 2015). Cell surface RNAs could activate endosomal TLRs upon phagocytosis. RNA N-glycosylation prevents detection by TLRs (Figure 2) by masking acp^3^U, a potent stimulator of TLR3 and TLR7 capable of inducing a type I IFN response during apoptotic cell clearance (Graziano et al., 2025). RNA de-N-glycosylation could therefore be a mechanism contributing to immunogenic cell death upon injury or infection. GlycoRNA associated with proteins and heparan sulfate appears to contribute to immune receptor binding, opening the door for glycoRNAs to contribute to immune responses (Li et al., 2025).

### 4.5 Changing appearances: adenosine-to-inosine editing

dsRNA is a potent immunostimulatory molecule, and safeguards are present to avoid pathological immune activation. The presence of endogenous dsRNA is largely prevented by the family of Adenosine Deaminase RNA Specific (ADAR) enzymes, which catalyze the deamination of adenosine (A) bases to inosines (I) (Bass and Weintraub, 1988; Lamers et al., 2019) (Figure 2), the most common base change in mammalian RNA (Gerber and Keller, 2001). A-to-I editing of dsRNAs leads to the formation of I-U wobble base pairs. Multiple sequential I-U base pairs destabilize dsRNA, lowering their immunogenic potential (Vitali and Scadden, 2010; Mannion et al., 2014). The majority of known A-to-I editing sites are found in short repetitive DNA sequences, the most abundant ones being *Alu* elements (Chen and Hur, 2022), comprising >10% of the human genome (Lander et al., 2001; Deininger, 2011). Two *Alu* elements transcribed in opposite orientation can form dsRNA species (Chen and Hur, 2022), with the major source of endogenous dsRNA estimated to be inverted repeat Alu elements (IR-Alu). IR-Alus are juxtaposed *Alu* elements in the same transcript that fold back and form long (∼300 bp) hairpins (Mehdipour et al., 2020). ADAR1 catalytic activity destabilizes immunostimulatory dsRNA species, offering protection from self RNA sensing (Sun et al., 2025). Mutations in *ADAR1* are causally associated with autoimmunity (Rice et al., 2012), underscoring the clinical relevance for a better understanding of self RNA recognition.

## 5 Erroneous self RNA sensing and immune pathology

RNA sensing by cellular PRRs triggers an inflammatory response that must be timely and limited (Takeuchi and Akira, 2007). Erroneous self RNA sensing thus leads to immune pathology. Gene mutations in *ADAR1* leading to erroneous self dsRNA sensing cause AGS (Rice et al., 2012; Liddicoat et al., 2015; Nakahama et al., 2021), a type-I interferonopathy characterized by IFN-induced brain injury and high childhood mortality rates (Liu and Ying, 2023). Likewise, MDA5 gain-of-function mutations can increase dsRNA binding avidity, particularly to *Alus*, causing an enhanced type-I IFN response and leading to AGS or systemic lupus (Rice et al., 2014; Ahmad et al., 2018). Gain-of-function mutations in TLR7 can cause lupus erythematosus (Brown et al., 2022). These mutations underscore the importance of maintaining self-RNA sensing at bay.

In mouse models, ADAR1 deletion is embryonically lethal (Hartner et al., 2004; Wang et al., 2004; Ward et al., 2011; Liddicoat et al., 2015), and ADAR1 mutations found in AGS patients induce a type-I IFN response (de Reuver et al., 2021; Guo et al., 2021; Inoue et al., 2021; Maurano et al., 2021; Nakahama et al., 2021; Tang et al., 2021; de Reuver et al., 2022; Hu et al., 2023). This is largely driven by the cytoplasmic IFN-inducible ADAR1 p150 isoform (Mannion et al., 2014; Pestal et al., 2015). In mice, AGS-related ADAR1 p150 mutations present in the catalytic or Zα protein domains drive aberrant IFN signaling by increased cellular dsRNA levels, causing erroneous MDA5 activation and downstream signaling via the adaptor protein mitochondrial antiviral signaling (MAVS) (Mannion et al., 2014; Liddicoat et al., 2015; de Reuver et al., 2021; Maurano et al., 2021; Tang et al., 2021; de Reuver et al., 2022). ADAR1 Zα mutations drive activation of the ISG and nucleic acid sensor ZBP1, causing ZBP1-mediated inflammation (de Reuver et al., 2022; Hubbard et al., 2022). It has recently been shown that not just ADAR1 p150 catalytic activity (Maurano et al., 2021) but also its dsRNA binding ability is necessary to suppress activation of the ISG and dsRNA sensor PKR, preventing a harmful inflammatory response (Hu et al., 2023). Ectopic expression of wild-type ADAR1 can rescue ISG expression *in vitro* (Liddicoat et al., 2015), opening the therapeutic potential of developing ADAR1-enhancing therapies. Importantly, the role of the ADAR1-dsRNA-MDA5 axis has recently been suggested as a shared mechanism underlying common inflammatory diseases (Li et al., 2022).

Cytosolic accumulation of dsRNA can also arise from mitochondria. Diminished PNPase protein levels cause increased cytosolic dsRNA (Dhir et al., 2018). Mutations in *PNPT1*, the gene encoding PNPase, are also associated with AGS (Bamborschke et al., 2021), making PNPase an interesting target for further research.

Although not directly linked with sensing, RNA can also contribute to the sex-bias existing in autoimmune conditions, predominantly affecting women. Autoantibodies against *XIST*-ribonucleoprotein complexes are only present in females since *XIST* is the long non-coding RNA that compensates X chromosome dosage (Dou et al., 2024).

Considering the large number of RNA-binding proteins (Yan et al., 2015), it is paramount to further understand self vs. non-self RNA binding, sensing, and processing. Investigating the mechanisms preventing self RNA sensing lays a foundation for better understanding immune pathologies, paving the way for novel treatment options, underscoring the need for research in this area.

## 6 mRNA as a therapeutic tool

*In vitro* transcribed mRNAs encoding physiologically and pathologically relevant proteins offer great therapeutic promise. Until the groundbreaking work by Karikó and Weissman, the clinical use of RNA remained unfeasible due to its labile and immunogenic nature (Sahin et al., 2014). Developing nucleoside-modified *in vitro* transcribed mRNAs that remained translatable while evading innate immune detection *in vivo* was revolutionary. Specifically, pseudouridine and N1-methylpseudouridine incorporation was found as a potent way to decrease RNA immunogenicity (Karikó et al., 2005; Karikó et al., 2008; Andries et al., 2015) while simultaneously increasing RNA stability and expression (Karikó et al., 2008; Andries et al., 2015).

As reviewed elsewhere (Nance and Meier, 2021; Verbeke et al., 2021; Liu and Wang, 2022; Leong et al., 2025), Karikó and Weissman laid the foundation for the development of the SARS-CoV-2 mRNA vaccines, decreasing mortality rates during the COVID-19 pandemic and saving an estimated 1.6 million lives in Europe alone (Meslé et al., 2024). Beyond direct protection from severe symptoms and death upon SARS-CoV-2 infection, the COVID-19 mRNA vaccines have beneficial side effects, such as sensitizing cancer patients to immune checkpoint blockade (Grippin et al., 2025). Moreover, the clear efficacy of mRNA vaccines developed during the COVID-19 pandemic opened the door to exploring mRNA-based therapeutics for other diseases, including various cancer types (Chen et al., 2024). The fundamental work elucidating basic principles underlying self vs. non-self RNA recognition led to novel RNA-based treatment strategies, which we believe will continue to emerge and transform therapeutics.

## 7 Concluding remarks

RNA sensing is a double-edged sword: while it allows for the recognition of viral RNAs (Luan et al., 2024), erroneous self RNA sensing causes incurable immune pathologies (Rice et al., 2012; Mannion et al., 2014; Liddicoat et al., 2015). A more comprehensive understanding of molecular safeguards against self RNA sensing is key to addressing this clinical need.

Fundamental work revealing RNA modifications as a means to distinguish self vs. non-self RNA (Karikó et al., 2005) opened a new research field, with its most recent addition being glycoRNAs (Flynn et al., 2021). Only a handful of other chemical RNA modifications and base changes have been implicated in self vs. non-self RNA recognition so far. With over 170 known RNA modifications (Sordyl et al., 2026), an untapped potential remains in exploring the role of RNA modifications in immune-mediated pathologies, antiviral responses, and antiviral therapies.

Understanding basic principles underlying self vs. non-self RNA recognition has been essential for mRNA-based therapeutics that allow the translation of physiologically and pathologically relevant proteins in different contexts, such as the SARS-CoV-2 mRNA vaccines (Nance and Meier, 2021; Verbeke et al., 2021; Liu and Wang, 2022; Leong et al., 2025). Further investigating the roles and consequences of other RNA modifications in RNA recognition and lifespan will undoubtedly broaden the current therapeutic potential of RNA.

In summary, the many facets of RNA and its adequate sensing are essential for immune balance and antiviral responses, and open new avenues in the emerging field of RNA-based therapies.
